# Talking Cure Models: A Framework of Analysis

**DOI:** 10.3389/fpsyg.2017.01589

**Published:** 2017-09-13

**Authors:** Christopher Marx, Cord Benecke, Antje Gumz

**Affiliations:** ^1^Department of Psychosomatics and Psychotherapy, Psychologische Hochschule Berlin Berlin, Germany; ^2^Department of Psychology, University of Kassel Kassel, Germany; ^3^Department of Psychosomatic Medicine and Psychotherapy, University Medical Center Hamburg-Eppendorf and Schön Clinic Hamburg Eilbek Hamburg, Germany

**Keywords:** talking cure, language in psychotherapy, verbal interventions, theoretical framework, common factors

## Abstract

Psychotherapy is commonly described as a “talking cure,” a treatment method that operates through linguistic action and interaction. The operative specifics of therapeutic language use, however, are insufficiently understood, mainly due to a multitude of disparate approaches that advance different notions of what “talking” means and what “cure” implies in the respective context. Accordingly, a clarification of the basic theoretical structure of “talking cure models,” i.e., models that describe therapeutic processes with a focus on language use, is a desideratum of language-oriented psychotherapy research. Against this background the present paper suggests a theoretical framework of analysis which distinguishes four basic components of “talking cure models”: (1) a *foundational theory* (which suggests how linguistic activity can affect and transform human experience), (2) an *experiential problem state* (which defines the problem or pathology of the patient), (3) a *curative linguistic activity* (which defines linguistic activities that are supposed to effectuate a curative transformation of the experiential problem state), and (4) a *change mechanism* (which defines the processes and effects involved in such transformations). The purpose of the framework is to establish a terminological foundation that allows for systematically reconstructing basic properties and operative mechanisms of “talking cure models.” To demonstrate the applicability and utility of the framework, five distinct “talking cure models” which spell out the details of curative “talking” processes in terms of (1) *catharsis*, (2) *symbolization*, (3) *narrative*, (4) *metaphor*, and (5) *neurocognitive inhibition* are introduced and discussed in terms of the framework components. In summary, we hope that our framework will prove useful for the objective of clarifying the theoretical underpinnings of language-oriented psychotherapy research and help to establish a more comprehensive understanding of how curative language use contributes to the process of therapeutic change.

## Introduction

Psychotherapy has traditionally been considered as a “talking cure” (Breuer and Freud, 1895/2001 p. 30), a treatment method that operates through an “exchange of words.” (Freud, 1916-17/1963, p. 17). Over the decades this notion proved to be problematic: On the one hand, it was demonstrated that therapeutic processes are decisively shaped by non-verbal aspects of interaction, e.g., facial expression (Benecke et al., 2005), synchronization of body movement (Ramseyer and Tschacher, 2011), or speech pauses (Levitt, 2002). On the other hand, psychotherapy research in terms of empirically supported treatments (Chambless and Ollendick, 2001), common factors (Laska et al., 2014), specific therapeutic techniques (Tschacher et al., 2014), principles of therapeutic change (Castonguay and Beutler, 2006), or helpful events (Timulak, 2007) established that therapeutic change is achieved through a multiplicity of factors, most of which cannot be reduced to language use.

Despite these differentiations, many researchers insist on the paramount importance of language use in therapeutic processes. Recent approaches describe “talk” as basic “means of delivery” of psychotherapy (Wampold, 2007, p. 860), “communicative exchange” as “substance of the clinical process” (Salvatore and Gennaro, 2015, p. 197), or emphasize that the “very idea” of psychotherapy is “based on the assumption that it is possible for one person to resolve a problem through talking to another” (Symington, 2006, p. 2; see also Russell, 1989; Frank and Frank, 1993; Stiles, 2017). Such positions are further underscored by contributions from disciplines like philosophy, linguistics, semiotics, or communication theory which emphasize that human existence is fundamentally shaped by language: “Language so permeates how we come to take persons, processes, and things that it is impossible to imagine how things might be outside and untouched by our linguistically mediated construals” (Russell, 1989, p. 509). In particular, various functions of language were established, e.g., to describe the “world” (*representational function*; Russell, 1989), to transport information from sender to receiver (*communicative function*; Shannon and Weaver, 1963), to articulate thoughts or feelings and situate them in a public space (*expressive function*; Taylor, 2016), to perform acts through speech (*performative function*; Austin, 1975), to prompt specific reactions in the receiver (*conative function*; Jakobson, 1990), or to initiate and maintain social intercourse (*phatic function*; Malinowski, 1989).

In view of these considerations it appears essential to clarify the specific effects and mechanisms of language use in therapeutic processes. The question, then, is how “talking” can “cure,” and how such curative effects relate to the complex multiplicity of change-related factors in psychotherapy. At present, language-oriented psychotherapy research employs two broadly definable approaches to answer this question. Both are based on verbal data, mostly transcripts from therapy sessions. The first approach relies on quantitative methods and strives for description or evaluation of effects of verbal (inter-)actions in psychotherapy. For this purpose, rating systems are developed that permit coding of verbal data with reference to different objectives (Gumz et al., 2015), e.g., identifying specific types of verbal units (e.g., narratives; Angus et al., 2017), distinguishing different types of verbal units (e.g., verbal response modes; Stiles, 1992), or rating the degree to which specific verbal units are present in a given data set (e.g., intervention techniques; Gumz et al., 2017). Resulting categorical or metric data can be quantitatively processed, e.g., by correlation with outcome or process variables to assess their effects. The second approach employs qualitative methods to analyze linguistic aspects of psychotherapy based on different methodological paradigms like conversation analysis, narrative analysis, or discourse analysis (Georgaca and Avdi, 2009). Related studies focus on the description of verbal micro-processes, e.g., formulations of interpretations by psychoanalysts (Peräkylä, 2004), establishment of common ground between patient and therapist (Buchholz, 2016), or pathological narratives (Dimaggio and Semerari, 2001). Other studies have critical purposes, e.g., how power relations in therapeutic interactions reshape patients' perceptions of and relations to their problems (Hodges, 2002). The major advantage of qualitative approaches is their ability to specify fine-grained details of linguistic processes in psychotherapy that cannot be matched by quantitative rating systems. This comes at the cost of substantial effort of data-analyses, difficulties in meeting quality criteria of psychotherapy research, and limited possibilities of evaluating and generalizing findings due to small samples (Levitt et al., 2017).

An essential problem shared by both approaches of language-oriented psychotherapy research is the huge diversity of constructs investigated in associated studies. The “talking”-aspect of the “talking cure,” for example, has been defined terms of verbal interventions, verbalizations of emotions, narratives, metaphors, speech acts, discourse, conversation, and many more, while the “cure”-aspect of language-oriented research can refer to constructs like symptom reduction, catharsis, symbolization, self-coherence, experiencing, emotional intensity, and so forth. Accordingly, there is a multitude of findings and theories regarding diverse operative specifics of curative “talking” processes in psychotherapy. How these relate to each other and in what way they can be integrated and systematized, however, is rarely even problematized. In consequence, the umbrella term of the “talking cure” is as obvious as it is vague: While therapeutic processes are essentially based on a wide range of linguistic activities, the core principles and mechanisms of curative “talking” processes in therapeutic contexts remain unclear.

Against the background of this confusing state of affairs we propose that language-oriented psychotherapy research can profit from a clarification of its basic theoretical foundations. For this purpose, the present paper suggests a theoretical framework of analysis that can be used to systematically describe the theoretical structure of models of curative language use in therapeutic contexts. In the following we will refer to such models as “talking cure models” which we define as theoretical models that comprise (1) a “talking”-aspect that specifies particular types of linguistic (inter-)actions as central means of therapeutic agency, and (2) a “cure”-aspect that specifies particular types of curative change effects. Important to note: In our understanding the term “talking cure model” can refer to comprehensive psychotherapy models as well as circumscribed models of processes or interventions that are situated within such comprehensive models. In particular, the framework comprises four components: (1) A *foundational theory*, (2) an *experiential problem state*, (3) a *curative linguistic activity*, and (4) a *change mechanism*. The first part of the paper explicates these components, while the second part applies the framework to five “talking cure models” that spell out curative “talking” in therapeutic contexts in terms of (1) *catharsis*, (2) *symbolization*, (3) *narrative*, (4) *metaphor*, and (5) *neurocognitive inhibition* in order to illustrate its utility. In summary, we suggest that our framework can serve as heuristic tool that helps to analyze and systematically reconstruct the basic theoretical architecture and working principles of “talking cure models” which can, in turn, contribute to an integration of empirical findings of language-oriented psychotherapy research and thereby to a more comprehensive understanding of “language” as central factor within a multitude of disparate factors of therapeutic change. Furthermore, we hope that our paper can help to stimulate further research on theoretical underpinnings of therapeutic processes in general which is, according to a growing number of researchers, a fundamental desideratum of contemporary psychotherapy research (Kazdin, 2001; Stiles et al., 2015; Gelo and Salvatore, 2016).

## The architecture of “talking cure models”: a theoretical framework of analysis

Below we present a theoretical framework of analysis that is supposed to describe the basic theoretical structure of “talking cure models” in dyadic settings, i.e., models that conceptualize curative therapeutic processes in terms of linguistic (inter-)action between patient and therapist. The framework emerged in an extensive process of reviewing literature on the “talking cure,” i.e., classic theoretical texts (e.g., Gendlin, 1997; Freud, 1915/1957) as well as empirical contributions (Greenberg, 1986; Russell, 1989) in terms of rating-systems (Gumz et al., 2015) or qualitative research (Georgaca and Avdi, 2009). The objective of this review was to understand and systematize the theoretical “architecture” of such models, i.e., how they are *constructed* and how they conceptualize the *operative specifics* of curative “talking” processes. In this section, we will briefly outline the framework components, while the next section will illustrate the application of the framework with regard to five “talking cure models.” Both sections are dialectically intertwined: The framework will be used to discuss the “talking cure models” in the next section; at the same time these models are part of a far greater number of models that were used to construct the framework as presented in this section. Accordingly, the considerations in the present and the following section represent stages in a hermeneutic process (Gadamer, 2013) that is open to further elaboration.

In general, we suggest to describe the procedural logic of “talking cure models” as follows: Some kind of linguistic activity aims at some kind of experiential problem state of the patient and transforms the problem state in a curative way. For purposes of clarification we distinguish four components within this formula: (1) a foundational theory, (2) an experiential problem state, (3) a curative linguistic activity, and (4) a change mechanism. The following sections explicate these components which are summarized in Table 1.

**Table 1:** Components of the theoretical framework of analysis for “talking cure models.”

**Framework components**	**Descriptions**	**Abbreviations**
**1. Foundational theory**	Theory that specifies general assumptions regarding function(s) of language (use) and its effects on human experience that are applied to therapeutic context	FOUND-THEORY
**2. Experiential problem state**	Defines the problem/pathology (“why”) of patient that is supposed to be “cured” by linguistic activity	
Experiential unit(s)	Dysfunctionally organized aspect(s) of experience that constitute/s problem/pathology of patient	EXP-UNIT
Experiential focus	Scope of experiential unit(s)	
Specific	Single or narrowly defined class of experiential unit(s)	>SPEC
Global	Broadly defined or generic experiential unit(s)	>GLOB
Problem-language-relation	Relation of problem/pathology of patient to linguistic unit(s)	P-L-REL
Lack of language	Problem/pathology is due to incapacity to find linguistic unit(s) that represent experiential unit(s)	>LACK
Expressed linguistically	Problem/pathology is represented by linguistic unit(s)	>EXPR
**3. Curative linguistic activity**	Defines agent(s) (“who”) and means (“what”) of curative linguistic activity	
Speaker position	Agent(s) of curative linguistic activity	SP-POS
Patient	Patient is agent of linguistic activity	>PAT
Therapist	Therapist is agent of linguistic activity	>THER
Patient/Therapist	Patient and therapist are agents of linguistic activity and contribute equally to linguistic activity	>PAT/THER
Patient + Therapist	Patient and therapist are agents of linguistic activity and patient has leading role	>PAT+THER
Therapist + Patient	Patient and therapist are agents of linguistic activity and therapist has leading role	>THER+PAT
Linguistic unit(s)	Linguistic activity that affects and curatively transforms experiential problem state	LING-UNIT
Linguistic focus	Scope of linguistic unit(s)	
Specific	Single or narrowly defined class of linguistic unit(s)	>SPEC
Global	Broadly defined or generic linguistic unit(s)	>GLOB
**4. Change mechanism**	Defines processes and effects (“how”) of curative linguistic activity	
Process(es)	Process(es) by which linguistic activity curatively transforms experiential problem state	CM-PROC
Effect(s)	Effect(s) generated by transformational process(es)	CM-EFF
Primary effect(s)	Primary effect(s) of transformational process(es)	>PRIM
Secondary effect(s)	Effect(s) generated by primary effect(s) of transformational process(es)	>SEC

### Foundational theory

The *foundational theory* is the backbone of any “talking cure model.” In most cases, these theories derive from extra-clinical contexts (e.g., philosophy, linguistics, semiotics, or communication theory) and have *per se* no clinical implications but specify general function(s) of language (use), e.g., to clarify cognitions, communicate with other persons, or perform actions. “Talking cure models” adopt (parts of) such theories and apply them to clinical contexts. Formally stated, this adoption can be described as a merging of the extra-clinical theory with clinical knowledge and procedures with the purpose of devising a model that explains how the problem or pathology of the patient can be affected and transformed by specific linguistic activities by means of specific processes that generate specific effects. The particulars of this process are explicated by the following three components.

### Experiential problem state

The *experiential problem state* defines the “why” of the “talking cure model,” i.e., the problem or pathology of the patient that needs to be “cured” by linguistic activity. In particular, it specifies one or more dysfunctionally organized *experiential unit(s)* that constitute/s the problem/pathology as well as the aim(s) of curative linguistic activity. If the problem state specifies more than one experiential unit, these can be organized in a sequence which suggest an “etiology” of the problem/pathology (e.g., trauma generates aversive affect>aversive affect cannot be abreacted>hysteric symptoms). As the experiential units specified in “talking cure models” range substantially regarding their scope, we distinguish a global vs. specific *experiential focus*: A *specific experiential focus* refers to a single or narrowly defined class of experiential units (e.g., affect), while a *global experiential focus* refers to broadly defined or generic experiential units (e.g., traumatic events). Furthermore, the problem state definition explicitly or implicitly indicates the relation of the problem/pathology to linguistic units. In our review process two possible *problem-language-relations* emerged: Either the problem state is due to a *lack of language*. In this case, the pathology is conceived as being caused by an incapacity to *find* or *express* a specific experiential unit (e.g., anger affect) by adequate linguistic units (e.g., the word “anger”), while the “cure” of this pathology consists in *finding* such linguistic units (e.g., the statement “I am angry”). Or the problem state is *expressed linguistically*, i.e., the pathology is *represented* by linguistic units (e.g., a metaphor like “I am a loser” which represents the patient's self-conception) which implies that the “cure” consists in therapeutically targeting and reorganizing this linguistic unit (e.g., differentiating the “loser”-metaphor) which curatively retroacts to the pathology.

### Curative linguistic activity

The *curative linguistic activity* defines the “who” and “what” of the “talking cure model.” The “who” is defined by the *speaker position* which specifies the agent(s) of curative “talking” processes. In dyadic settings three positions are possible: Either the patient is “talking,” or the therapist, or both. Within the third position further differentiations are possible, e.g., when a model considers both patient's and therapist's linguistic activities while attributing the leading role to one party. The “what” of the “talking cure” is defined by specifying one or more *linguistic unit(s)* that are assumed to affect and curatively transform the patient's experiential problem state. If it specifies more than one linguistic unit, these can be organized in different sequences (e.g., linear sequence, reciprocally progressing sequence, etc.). As the linguistic units specified in “talking cure models” range substantially regarding their scope, we distinguish a global vs. specific *linguistic focus*: A *specific linguistic focus* refers to a single, narrowly defined class of verbal activities (e.g., interpretation), while a *global linguistic focus* refers to broadly defined or generic verbal activities (e.g., narrative).

### Change mechanism

The *change mechanism* specifies “how” linguistic activities curatively affect and transform the patient's experiential problem state. For this purpose it defines transformational *process(es)* effectuated by linguistic activities (e.g., expulsion of “strangulated affect”) as well as *effect(s)* of such processes (e.g., cathartic purgation). If the model specifies more than one effect, these can be organized in a sequence with *primary effects* (e.g., cathartic purgation) and *secondary effects* (e.g., dissolution of hysteric symptoms).

## Models of the “talking cure”

In order to illustrate the applicability and utility of the theoretical framework, the following sections discuss five “talking cure models” which spell out curative “talking” processes in different ways, i.e., in terms of (1) *catharsis* (Breuer and Freud, 1895/2001) (2) *symbolization* (Freud, 1915/1957), (3) *narrative* (Schafer, 1992) (4) *metaphor* (Buchholz, 2007), and (5) *neurocognitive inhibition* (Lieberman et al., 2007). Important to note: By selecting these models we are *not* attempting to constitute an exhaustive classification of “talking cure models.” Rather, the models were selected with regard to their relevance for past and present debates in language-oriented psychotherapy research. In summary, they represent the complex multitude of thinking the operative principles of curative language use in psychotherapy. For each model we provide an introduction to its theoretical tradition, then outline its central idea of the “talking cure,” and finally summarize the model in terms of the framework components using the abbreviations defined in Table 1. An overall synopsis of the model discussions is presented in Table 2.

**Table 2:** Discussion of five “talking cure models” in terms of the theoretical framework of analysis.

**Talking cure models**	**1. Foundational theory**	**2. Experiential problem state**				**3. Curative linguistic activity**			**4. Change mechanism**			
		**Experiential unit(s) (Focus)**		**Problem-language-relation (Focus)**		**Speaker position**		**Linguistic unit(s) (Focus)**		**Process**		**Effect(s)**
Catharsis model (Breuer and Freud)	Catharsis theory (e.g., Scheff, 2001)	Traumatic event (G) ↓ “Strangulated affect” (S) ↓ Hysteric symptoms (G)	→	Lack of language: “Strangulated affect” cannot be verbalized (S)	→	P ↓ P	→ →	Re-telling of traumatic event by patient (G) ↓ Verbalization of “strangulated affect” by patient (S)	→	Expulsion of “strangulated affect” from “inside” to “outside”	→	Cathartic purgation ↓ Dissolution of hysteric symptoms
Symbolization model (Freud; Loewald; Lorenzer)	Neurological theory of thing- and word-presentation (e.g., Freud, 1891/1953)	Negative relational experiences in early life (G) ↓ Unconscious representations of negative relational experiences (thing-presentations) (G) ↓ Maladaptive interactional repertoire (G)	→	Lack of language: Representations of relational experiences cannot be verbalized (G)	→	T	→	Interpretations by therapist (S)	→	Symbolic linking of representations of relational experiences with language (thing- with word-presentations)	→	Relational experiences become conscious ↓ Distancing; Weakening of action-impulses; Planning; Trial-Acting ↓ More adaptive interactional repertoire
Narrative model (Schafer)	Narrative theory (e.g., Schafer, 1992)	Pathological organization of life-, self-, and identity conceptions (G)	→	Expressed linguistically: Dysfunctional maladaptive, incoherent, or disrupted life-, self-, and identity narrative represents patient's pathology (G)	→	P ↕ T	→ →	Telling of life-, self-, and identity narrative by patient (G) ↕ Punctual modifying, correcting, and reshaping of the patient narrative through interpretations by therapist (S)	→	Gradually progressing narrative reorganization of pathological life-, self- and identity narrative	→	More complete, coherent, convincing and adaptively useful life-, self-, and identity narrative ↓ More adaptive organization of life-, self-, and identity conceptions
Metaphor model (Buchholz)	Conceptual metaphor theory (e.g., Lakoff and Johnson, 2003)	Pathological organization of conceptions of self, symptoms, and relations to others; Basic human conflicts or paradoxes (G)	→	Expressed linguistically: Dysfunctional metaphor represents patient's pathology, conflicts or paradoxes (S)	→	T+P ↓ T+P	→ →	Analyzing the dysfunctional metaphor by therapist and patient (G) ↓ Changing the dysfunctional metaphor by therapist and patient (S)	→	Breaking-up of maladaptive source-target conjunctions and establishing link of target-domain with more adaptive source-domain	→	More beneficial, adaptive metaphors ↓ More adaptive organization of conceptions of self, symptoms, and relations to others; Manageability of basic conflicts or paradoxes; Individual growth
Inhibition model (Lieberman)	Neurocognitive theory of symbolic processing of affective stimuli (e.g., Lieberman et al., 2007)	Unregulated aversive affect (S) ↓ Emotional distress (G)	→	Lack of language: Aversive affect is not verbalized (S)	→	P	→	Verbalization of aversive affect by patient (S)	→	Inhibition due to a neurocognitive mechanism through which an increase in RVLPFC activity inhibits amygdala activity	→	Reduction of emotional intensity ↓ Alleviation of emotional distress

*Linguistic and experiential focus: S, Specific focus; G, Global focus. Speaker position: P, Patient; T, Therapist. RVLPFC, Right ventro-lateral prefrontal cortex*.

### Catharsis

The first model of the “talking cure” is centered around the idea of *catharsis* (Greek: *kátharsis* = “purgation”). Catharsis was initially associated with the dramatic theory of Aristotle‘s *Poetics* where it marks the social objective of the dramatic genre of tragedy which consists in the purgation of aversive affects in the audience (Scheff, 2001). In therapeutic contexts, the catharsis concept was introduced by Breuer and Freud in their *Studies on Hysteria* (Breuer and Freud, 1895/2001). In essence, the *Studies* assume that hysteria is caused by a traumatic event (e.g., sexual abuse) which generates an aversive affect that cannot be abreacted. In consequence, the affect is “*strangulated*” (Breuer and Freud, 1895/2001, p. 17), i.e., emotional reactions are repressed which in turn generates hysteric symptomatology. In order to treat these symptoms, a process of “talking” by the patient is necessary. Breuer and Freud describe this process as follows:

For we found, to our great surprise at first, that *each individual hysterical symptom immediately and permanently disappeared when we had succeeded in bringing clearly to light the memory of the event by which it was provoked and in arousing its accompanying affect, and when the patient had described that event in the greatest possible detail and had put the affect into words*. Recollection without affect almost invariably produces no result. The psychical process which originally took place must be repeated as vividly as possible; it must be brought back to its *status nascendi* and then given verbal utterance (Breuer and Freud, 1895/2001, p. 6).

This passage constructs the “talking cure” as a bipartite process. First, “talking” implies a detailed re-telling of the traumatizing event that has to be brought “clearly to light,” “in the greatest possible detail.” Plain re-telling, however, is not enough. As a second step, this re-telling needs to go along with a re-arousal of the affect that was constrained from being abreacted beforehand, and this process needs to culminate in a verbalization of the affect: It has to be “put into words,” “given verbal utterance”—otherwise the hysteric symptoms are not “cured.” A little later in the text, this aspect is formulated more precisely when the authors summarize the operating principle of their “therapeutic procedure”: “*It brings to an end the operative force of the idea* [i.e., the representation of the traumatic event] *which was not abreacted in the first instance, by allowing its strangulated affect to find a way out through speech*” (Breuer and Freud, 1895/2001, p. 17). This formulation clarifies the processual logic of the catharsis concept: Initially, there is a pathogenic agent on the “inside” of the patient, i.e., a non-abreacted, “strangulated” affect^1^. The cathartic “talking cure” then operates by verbalizing this affect, allowing it “to find a way out.” By consulting the original German text of the passage, this understanding can be elaborated. Here, the authors use the formulation “Ablauf gestatten” to specify what “speech” does to the affect (Breuer and Freud, 1895/2007, p. 40). In German, “ablaufen” refers to the flowing-off of a fluid, and “gestatten” means “to permit.” In other words, the non-abreacted affect is metaphorically rendered as a retained fluid which can flow off through verbalization. The “talking cure,” then, is a process in which the patient's “talking” removes the blockage of a pathogenic affect, resulting in its transportation from the “inside” to the “outside” and, eventually, a cathartic purgation—and this is what “cures” the patient from hysteric symptoms.

To summarize: The catharsis model according to Breuer and Freud is grounded in the classic notion of catharsis theory which posits that a focused arousal of aversive affects entails cathartic purgation (Scheff, 2001) [FOUND-THEORY]. Against this background, the model defines the experiential problem state as result of a lack of language [P-L-REL>LACK], i.e., in terms of hysteric symptoms [EXP-UNIT>GLOB] deriving from traumatic events [EXP-UNIT>GLOB] which generate an aversive affect that cannot be abreacted as it cannot be verbalized and which is therefore “strangulated.” [EXP-UNIT>SPEC] The curative verbal activity, then, is devised as a bipartite consecutive process in which the patient [SP-POS>PAT] first reproduces the traumatic event [LING-UNIT>GLOB] and then verbalizes the “strangulated affect.” [LING-UNIT>SPEC] This, in turn, effectuates the expulsion of the “strangulated affect” from the “inside” of the patient to the “outside” [CM-PROC], thereby entailing a cathartic purgation [CM-EFF>PRIM] and finally a dissolution of hysteric symptoms [CM-EFF>SEC].

### Symbolization

The second model of the “talking cure” describes psychotherapy in terms of symbolization (Greek: *symbolon* = “sign,” “token,” or “identification mark”). We will confine ourselves to a central notion of symbolization in terms “dynamic schematizing activity” (Werner and Kaplan, 1963) and focus on Freud's theory of symbolization.

Freud's symbolization theory is centered around the concepts of thing-presentation (“Sachvorstellung”) and word-presentation (“Wortvorstellung”) (Freud, 1891/1953, 1915/1957, 1923/1960). Both refer to different types of memory traces, i.e., representations of past perceptions (Erdelyi, 1985). Word-presentations are clearly defined as “derived primarily from auditory perceptions” (Freud, 1923/1960, p. 13), i.e., memories of heard language that constitute the speech apparatus (Freud, 1891/1953; see Forrester, 1980). For thing-presentations, the case is less clear, mainly due to a complex, often arbitrary occurrence of different but presumably synonymously used terms in Freud's writings (Rizzuto, 2015). The analytic tradition interprets the concept in terms of relational experience, i.e., as cognitive-affective schemata deriving from interactions with significant other persons that exert strong influence on the individual's behavior. Related concepts have been formulated by different authors, e.g., internal working models (Bowlby, 1969), representations of interactions that have been generalized (RIGs; Stern, 1985), emotion schemas (Bucci, 2007), or the introject surface of Benjamin's (1974) structural analysis of social behavior (SASB).

The substantial maladaptive consequences of negative relational experiences (thing-presentations) can be illustrated by the following example: Consider a patient with a history of frustrating interactions with his parents who tends to react to contemporary frustrations by parental figures (e.g., his boss) with massive outbursts of anger with according consequences (e.g., being fired). While being aware of the unfavorable consequences of his aggressive behavior, the patient cannot understand his outbursts—as he cannot make the connection to the driving force of his reaction, i.e., experiences with his parents. To outline the therapeutic task in this context with reference to Freud's symbolization model we use a passage from his meta-psychological paper on “The Unconscious” (Freud, 1915/1957):

The conscious presentation comprises the presentation of the thing plus the presentation of the word belonging to it, while the unconscious presentation is the presentation of the thing alone. The system *Ucs*. contains the thing-cathexes of the objects, the first and true object cathexes; the system *Pcs*. comes about by this thing-presentation being hypercathected through being linked with the word presentations corresponding to it. It is these hypercathexes, we may suppose, that bring about a higher psychical organization and make possible for the primary process to be succeeded by the secondary process which is dominant in the *Pcs*. (Freud, 1915/1957, p. 201f.).

This passage is centered around a linking operation, i.e., the linking of thing-presentations (also: “thing-cathexis,” see Loewald, 2000) and word-presentations. This operation is also referred to as “hypercathexis”: The (unconscious) thing-presentation is “hypercathected” by being linked to a (preconscious) word-presentation (Loewald, 2000). In his early writings on aphasia, Freud still used another term—symbolization (Freud, 1891/1953): When thing- and word-presentation are linked, the “word” *symbolizes* the “thing.” The result of this linking process is a “higher psychical organization” which is paralleled with the progression from unconsciousness (“the presentation of the thing alone”) to consciousness (“the presentation of the thing plus the presentation of the word belonging to it”) and from primary process to secondary process. In summary, the (symbolic) linking of thing-presentation and word-presentation is the precondition for conscious secondary process mentation. This progression, in turn, has behavioral consequences, as conscious mentation goes along with a distancing from impulses towards acting-out relational patterns that are condensed in thing-presentations, opening up potentials for action planning and trial-acting (Lorenzer, 2002).

The concepts of thing- and word-presentations have implications regarding the psychoanalytic notions of repression and interpretation. Repression is generally considered as a process in which (1) something unconscious is prevented from becoming conscious or (2) something conscious becomes unconscious (Freud, 1936/1979). In terms of thing- and word-presentation this process can be rendered more precisely: If consciousness depends on the linking of thing- and word-presentation, the operative target of the repression mechanism must be this link, i.e., repression either (1) prevents the establishment of a link between thing- and word-presentation (Freud, 1915/1957), or (2) severs already existing links (Loewald, 2000) which has been described as de-symbolization (Lorenzer, 2002). In this view, a specific understanding of analytic interpretations emerges: If it is the general function of interpretations to make the Unconscious conscious (Gumz et al., 2017), the theory of thing- and word-presentations allows for operationalizing this process as either (1) establishing a beforehand non-existing link or (2) re-establishing a severed link between thing- and word-presentation. The first notion has been hinted at by Freud's statement that making the Unconscious conscious “is done by supplying *Pcs*. intermediate links” between thing- and word-presentations “through the work of analysis” (Freud, 1923/1960, p. 14). The second notion was suggested by Loewald: “The analyst‘s interpretation, which translates the patient‘s unconscious (thing-) presentation into words originally belonging to it, helps to re-establish […] the […] bond between thing and word” (Loewald, 2000, p. 183). With reference to the patient from before, these considerations imply the following therapeutic task: First, the connections between recent maladaptive behavior (anger outbursts) and past relational experiences (frustrations by parents) need to be made conscious. This is achieved by means of interpretations of the therapist which link thing-presentations (relational experiences) to adequate word-presentations (i.e., words which represent these experiences). Gaining consciousness regarding the causes for his reactions, in turn, weakens impulses toward acting them out, allows for action planning and trial acting and altogether opens up possibilities for a more adaptive interactional repertoire.

To summarize: The symbolization model according to Freud is grounded in Freud's (1891/1953) neurological theory of thing- and word-presentations which posits that an individual's interactional repertoire is shaped by early relational experiences (thing-presentations as cognitive-affective schemata) that are unconscious but can be made conscious by being connected with words (word-presentations) that represent these experiences [FOUND-THEORY]. Against this background, the model defines the experiential problem state as result of a lack of language [P-L-REL>LACK], i.e., in terms of representations of dysfunctional relational experiences that emerge in the course of negative developmental processes and determine the individual's interactional repertoire in maladaptive ways because they remain unconscious as they cannot be verbalized [EXP-UNIT>GLOB]. The curative verbal activity, then, is devised as process in which the therapist [SP-POS>THER] establishes symbolic links between corresponding thing- and word-presentations [CM-PROC] by means of interpretations [LING-UNIT>SPEC]. This, in turn, effectuates the dysfunctional relational experiences to become conscious [CM-EFF>PRIM] which allows the patient to distance himself from the action impulses deriving from these experiences and opens up possibilities for planning and trial-acting and an altogether more adaptive interactional repertoire [CM-EFF>SEC].

### Narrative

The third model of the “talking cure” describes curative effects of “talking” in terms of narrative processes (Latin: *narrare* = “to tell”). Starting point for this approach are two basic premises, an anthropological and a cultural premise. The anthropological premise claims that the construction of narratives is a basic human activity (Boyd, 2009). From this point of view, man is a “*homo fabulans*” (Howard, 1991), a “story-telling animal” (Gottschall, 2013). The basic function of narrative is to organize life events, actions, embodied feelings, beliefs, intentions etc. in a linear and coherent way, thereby giving form and structure to otherwise contingent, disparate experiences. In this sense, narrative is a “core organizing principle of human consciousness that shapes lived experiences into personal stories” (Angus, 2012, p. 369). In other words, humans use narratives to make sense of their lives, to gain a sense of self and identity. The person you are is the story you tell: “We seem to have no other way of describing lived time save in the form of narrative” (Bruner, 2004, p. 692). The cultural premise, on the other hand, emphasizes that narratives are not only individual constructions. Prior to each individual narrative process, each culture offers pre-exististing “narrative models […] available for describing the course of a life” (Bruner, 2004, p. 694) which constitute a reservoir each individual can use to construct his or her own story. At the same time, these individual constructions are themselves the basis for continuing the narrative chain.

For psychotherapy one particular aspect of narratives is important: The assumption that narratives are used as organizing template for individual self or identity conceptions does not guarantee that such constructions are stable. On the contrary, a person's life story is an intricate thing, “a privileged but troubled narrative” (Bruner, 2004, p. 693), subjected to crises and catastrophes. From this point of view an interesting perspective on psychopathology arises: If the construction of life, self and identity narratives can go awry, mental disorders can be interpreted as problematic, dysfunctional, maladaptive narratives that developed in the process of an individual's life story. Accordingly, psychotherapy can be seen as an attempt to reverse, modify, correct or adjust such problematic narratives, i.e., as a technique of “rebiographing” (Howard, 1991, p. 194), a chance for patients “to “re-author” their life-narrative—to construct a more complete, coherent, or hopeful story of “who they are” (Grafanaki and McLeod, 1999, p. 290). In the following we will focus our discussion on one specific approach from this tradition by Roy Schafer.

A basic intention of Schafer's writings is to reformulate psychoanalysis as a hermeneutic discipline that can be spelled out in terms of narrative. From this point of view, the analytical situation is considered as “series of narrative accounts of actions” (Schafer, 1980, p. 63) and psychoanalysis altogether as “narrational project” (1980, p. 83). Within this project, both patient and therapist are actively engaged, the “psychoanalytic dialogue” is enacted by “two agents, each narrating or telling something to the other […] along psychoanalytic lines” (Schafer, 1981, p. 30). The objective of this process is not to construct a certain “single, necessary, definitive account of a life history” but rather to help patients to transform their dysfunctional narratives “into others that are more complete, coherent, convincing, and adaptively useful than those they have been accustomed to constructing” (Schafer, 1980, p. 63). It is important to note how Schafer conceives of this narrational enterprise. The part of the patient is *to tell*—about himself, his past or central relations to other persons in his life. The part of the therapist is described in the following passage:

In making interpretations, the analyst retells these stories [i.e., the (dysfunctional) stories the patient has told]. In the retelling, certain features are accentuated while others are placed in parentheses; certain features are related to others in new ways or for the first time; some features are developed further, perhaps at great length. […] The analyst's retellings progressively influence the what and how of the stories told by analysands. […] The end product of this interweaving of texts is a radically new, jointly authored work or way of working. One might say that during the course of analysis, there develops a cluster of more or less coordinated new narrations, each corresponding to periods of intensive analytic work on certain leading questions (Schafer, 1981, p. 31f.).

According to Schafer, the basic activity of the therapist can be summarized as *re-telling* of the patient's stories. Regarding this re-telling, four aspects need to be emphasized: First, the operative logic of re-telling can be defined as re-*organization* process. When the therapist re-tells the patient's stories, he gives them a new shape by using different operations, e.g., accentuating some details, fading out others or constructing new or different relations between them. Second, this reorganizational process is not conceived as punctiform operation but as a sequence of iterative cycles which “progressively influence” and thereby re-shape the patient's stories. Third, Schafer relates these operations to the technique of interpretation, i.e., all reorganizational operations described above are executed by means of interpretive utterances: “Interpretations are redescriptions or retellings of action along the lines peculiar to psychoanalytic interest” (Schafer, 1980, p. 83). Fourth, this notion of iterative reorganization-by-interpretation clarifies the nature of the collaboration between patient and therapist. Although Schafer repeatedly stresses that new, more functional narratives are “jointly authored,” his approach assumes that the driving force in this process is the therapist who actively shapes the patient's stories by interpretations. This asymmetry is also documented in his description of the patient as the “coauthor of the analysis” (Schafer, 1981, p. 34)—which makes the therapist the “author” of the “narrational project.”

To summarize: The narrative model according to Schafer is grounded in narrative theory which posits that narrative is a central organizing principle of human experience that merges disparate elements of an individual's life (cognitions, emotions, memories, beliefs) into a linear story that constitutes a stable and coherent notion of life, self and identity (Schafer, 1992) [FOUND-THEORY]. Against this background, the model defines the experiential problem state as being expressed linguistically [P-L-REF>EXPR], i.e., as pathological organization of life-, self-, and identity conceptions that crystallize in a dysfunctional, maladaptive, incoherent, or disrupted life-, self-, and identity narrative which reflects the patient's pathology [EXP-UNIT>GLOB]. The curative verbal activity, then, consists in a two-sided process which is dominated by the therapist [SP-POS>THER+PAT]: The patient tells his story [LING-UNIT>GLOB] while the therapist intervenes into this process by means of punctual interpretations [LING-UNIT>SPEC]. In consequence, therapy can be interpreted as gradual process in which the narrations of the patient are iteratively modified, corrected, and reshaped by the therapist which leads to a gradually progressing narrative reorganization of the patient's narrative [CM-PROC] with the objective of finally constituting a more complete, coherent, functional, convincing, and adaptively useful narrative [CM-EFF>PRIM] and thereby to more adaptive conceptions of life, self and identity [CM-EFF>SEC].

### Metaphor

The fourth model of the “talking cure” is based on metaphor theory. Historically, metaphors were defined by Aristotle as an operation of *transference* (Greek: *metaphorein* = “to transfer”). For example, in the metaphor “Hercules is a lion,” the properties of strength and ferocity that are associated with lions are transferred to Hercules, implying that he is a strong and fierce fighter (Levin, 1982). The subsequent history of metaphor is shaped by two notions: First, it was assumed that metaphors are nothing but linguistic devices, rhetorical figures of speech. Second, metaphors were for the most part devalued as ornamental in function, “a sort of happy extra trick with words” (Richards, 1965, p. 90).

Both notions were overturned by metaphor theory of the 20th century. Building on the re-definition of metaphor in interactional terms (Black, 1954; Richards, 1965), Lakoff and Johnson (2003) formulated conceptual metaphor theory which established a new understanding of metaphor. They asserted that metaphoricity should not be treated as predominantly linguistic phenomenon but as a basic cognitive capacity of the mind which operates intrinsically metaphorically. In principle, what metaphorization allows for is to see one “thing” (or conceptual domain) in terms of another “thing” (or conceptual domain). This is achieved by projecting a specific set of attributes of one domain (= source domain) onto the other domain (= target domain). In most cases, the target domain is abstract while the source domain is concrete. This is why conceptual metaphors “make sense”: Seeing an abstract entity in terms of a concrete entity familiarizes the abstract entity, makes it more tangible and manageable. Precondition of this projection is a plausible set of shared characteristics of both domains. Consider Lakoff's (1986) classic example LOVE IS A JOURNEY. In this example, “JOURNEY” is the source domain from which specific characteristics are projected onto the target domain “LOVE.” This makes sense, as both domains share a set of common characteristics (e.g., traveler vs. lover, path of travel vs. path of relationship; Gibbs, 2011). Accordingly, the conceptual metaphor offers a specific perspective on what love “is.” The conceptual metaphor LOVE IS A TABLE, on the other hand, does not work, as both domains share no plausible characteristics. The conceptual metaphor LOVE IS WAR, in turn, makes sense again—but advances a completely different perspective on love than the “JOURNEY”-metaphor. Both are valid—and may be adequate depending on the circumstances of the person using it. A person happily engaged will consider LOVE IS A JOURNEY as adequate while a person in the middle of a divorce may prefer the WAR-metaphor. Neither perspective is “correct” as there are no definite conjunctions of domains (Gibbs, 2011).

In summary, these considerations imply that linguistic metaphors are a surface phenomenon, the reflection of a basic cognitive metaphorization activity (Gallese and Lakoff, 2005) that crystallized in a wide range of conceptual metaphors which can be found in almost any language throughout history in various domains of human culture (Gibbs, 2011). Important to note, however, is that conceptual metaphors cannot be reduced to condensations of cognitions. There is substantial evidence that metaphors are grounded in embodied experience and therefore have an affective-physiological component (Gibbs et al., 2004; Buchholz, 2007). Moreover, it needs to be emphasized that conceptual metaphors not only open up perspectives but also have limiting properties. For example, a person whose conception of intimate relationships is structured by the metaphor LOVE IS WAR may have difficulties to see positive aspects of relationships. In other words: Metaphors can also prevent a person from seeing something in differentiated ways.

There are a number of applications of metaphor theory in clinical contexts. Basic assumption of such applications is that metaphors have a lot to tell about people by representing central aspects of self-identity or interactional experience (Angus and Rennie, 1989) or central clinical topics or symptoms (Rasmussen and Angus, 1996). In the following we will focus our discussion on one approach towards therapeutically working with metaphors by Michael Buchholz.

Buchholz' starting point is a notion of human existence in terms of paradox: “What moves us are human paradoxes—contradictions that on a certain level appear to be inextricable” (Buchholz, 2007, p. 164). In dealing with such paradoxes linguistic metaphors have a central role, as they *articulate* paradoxes in a way no other verbal utterance could. In psychotherapy, now, a central focus lies on dysfunctional metaphors, i.e., metaphors that determine a patient's perception of himself, his relations to others or his symptoms in maladaptive ways. Consider the example of a patient longing for close, intimate relationships who at the same time reacts extremely aversive toward closeness and, accordingly, ends up conceiving himself as generally harmful in relationships. One way to express this self-conception may be the conceptual metaphor I AM POISON. Against the background of conceptual metaphor theory, this example would demonstrate the limiting potential of metaphors, as it expresses the general self-conception and behavior of a patient in intimate relationships. In other words, this patient is “in the grasp of [a] metaphor” (Buchholz et al., 2015, p. 893). The task of therapy, then, according to Buchholz, would be two-fold: First, to analyze the metaphor, and then “changing the metaphor when it becomes obsolete and outdated” (Buchholz, 2007, p. 167).

The operative target of the changing procedure can be stated in terms of conceptual metaphor theory. As outlined above, Lakoff and Johnson (2003) assume that there is no definite association between source and target domain in conceptual metaphors. This means that a target domain can receive its projections from different source domains (e.g., “JOURNEY” vs. “WAR”). This opens up an interesting perspective regarding the objective of metaphor-related therapeutic work, i.e., changing dysfunctional source-target-connections. What this implies can be illustrated for the patient from above. In his metaphor—I AM POISON—“POISON” is the source domain and “I” the target domain, i.e., the self (“I”) is constructed in terms of “POISON.” However, this conjunction of domains is not absolute, the “I” can also be constructed in more positive ways that accentuate resources of a person or beneficial aspects of its self (e.g., I AM A RESOURCEFUL PERSON). The purpose of therapy, then, would be working toward this end by iteratively loosen the ingrained association of the “I”-target from the “POISON”-source, e.g., by focusing therapeutic work on analyzing and elaborating cognitions or feelings that are associated with the “old,” dysfunctional, pathological conceptual metaphor with the purpose of helping the patient see himself in “new,” more adaptive terms and, thereby, making the paradoxical experience more manageable (Buchholz, 2007, p. 164). This objective Buchholz also described in terms of individual growth which is a “highly central potential in bearing unbearable conflicts” (Buchholz, 2015, p. 88). The details of such processes were discussed by Buchholz in a number of publications, more recently with a focus on transcript data that is analyzed using conversation analysis which demonstrated that change processes of conceptual metaphors not only occur on macroscopic levels, i.e., as final therapy result, but can also be observed on the micro-level of in-session interactions (Buchholz et al., 2015; Buchholz, 2016).

To summarize: The metaphor model according to Buchholz is grounded in conceptual metaphor theory which posits that metaphorization is a basic cognitive capacity that allows for familiarizing abstract concepts by means of metaphorically projecting properties of more concrete concepts onto the abstract concepts. Moreover, it interprets metaphors as linguistic representations of basic self-conceptions that are rooted in embodied experience (Lakoff and Johnson, 2003) [FOUND-THEORY]. Against this background, the model defines the experiential problem state as being expressed linguistically [P-L-REF>EXPR], i.e., as pathological organization of general conceptions of self, symptoms, or relations to others or basic human conflicts or paradoxes [EXP-UNIT>GLOB] that are linguistically represented by a metaphor [EXP-UNIT>SPEC]. The curative verbal activity, then, consists in a joint procedure of analyzing the metaphor [LING-UNIT>GLOB] where the therapist has the leading role [SP-POS>THER+PAT] and, if necessary, changing the metaphor [LING-UNIT>SPEC] in a process of breaking up maladaptive source-target conjunctions (e.g., I AM POISON) and establishing conjunctions of the target-domain (“I”) with a more adaptive source-domain (e.g., “RESOURCEFUL”) [CM-PROC]. This, in turn, leads to an availability of more adaptive and beneficial metaphors [CM-EFF>PRIM] which is assumed to go along with a more adaptive organization of conceptions of self, symptoms, or relations to others, an increased manageability of basic conflicts or paradoxes, and individual growth [CM-EFF>SEC].

### Neurocognitive inhibition

A final “talking cure model” originates from neurocognitive research on the effects of linguistic processing of emotional stimuli which has been used and evaluated in therapeutic contexts. We will outline this last model with reference to a study by Lieberman et al. (2007). The focus of this study was the question how verbalizations of emotions (“putting feelings into words”) may contribute to the alleviation of negative emotional responses. To investigate this question, participants of the study performed an affect labeling task: After looking at photos of faces with negative emotional expressions (fear or anger), they had to choose the correct verbal affect label from a pair of words shown below the face stimuli (e.g., “scared” vs. “angry”). At the same time, neurocognitive processing of the affect labeling task was assessed using functional magnetic resonance imaging. In their results, Lieberman and colleagues found an interesting effect pattern: First, they observed a significant reduction in the activity of the amygdala which is associated with emotion processing. This effect indicated that linguistic processing of emotional stimuli decreases the intensity of emotional experiences. Second, they found a significant increase in the activity in the right ventro-lateral prefrontal cortex (RVLPFC). As this region of the brain has been associated with symbolic processing of emotional information and with top-down inhibitory processes, Lieberman and colleagues hypothesized that an increase in RVLPFC-activity may inhibit amygdala activity. Further evidence suggested that this inhibitive process may be mediated by the medial prefrontal cortex (MPFC). In summary, the following mechanism emerges: When negative emotional stimuli are linguistically processed, the RVLPFC is activated which—mediated by the MPFC—inhibits the activity of the amygdala, thereby reducing the intensity of the negative emotional experience. In simple terms: Putting negative feelings into words helps to alleviate negative emotional responses by dampening experiential intensity.

Subsequent research extended the scope of these findings. First, a study by Lieberman et al. (2011) demonstrated that the neurocognitive mechanism outlined above also applies to positive emotional stimuli. This indicates that linguistic processing of emotions may generally entail an intensity reduction of emotional experience. Second, a study of Tabibnia et al. (2008) investigated the time-frame of such effects. In two experiments with negative emotional stimuli they observed that affect labeling tasks generated an attenuation of autonomic activity that was stable over the period of 1 week. This suggests that linguistic processing of emotional stimuli has not only immediate but also mid-term and possibly long-term effects on emotional reactivity. Finally, the efficacy of this neurocognitive mechanism was repeatedly corroborated in clinical contexts. It was found, for example, that affect labeling procedures significantly contributed to the effect of exposure treatment for spider-fearful persons (Kircanski et al., 2012) or persons with public speaking anxiety (Niles et al., 2015) which implies that the findings are not only relevant for experimental settings but also for real-life contexts.

To summarize: The inhibition model is grounded in a neurocognitive theory of symbolic processing of emotional stimuli which posits that verbalizations of affects lead to a reduction of affect intensity by means of a neurocognitive mechanism in which symbolic processing causes an increase in RVLPFC activity which inhibits the activity of the amygdala (Lieberman et al., 2007) [FOUND-THEORY]. Against this background, the model defines the experiential problem state as the result of a lack of language [P-L-REF>LACK], i.e., in terms of a non-regulated aversive negative affect [EXP-UNIT>SPEC] that is painfully intense as it is not verbalized. The curative verbal activity, then, consists in the verbalization of the affect [LING-UNIT>SPEC] by the patient [SP-POS>PAT] which leads to a reduction of its emotional intensity [CM-EFF>PRIM] and an alleviation of emotional distress [CM-EFF>SEC] by means of a neurocognitive inhibition mechanism [CM-PROC].

## Conclusion

Describing psychotherapy as a “talking cure” is self-evident and vague at the same time: Self-evident, as therapeutic processes are essentially based on language; and vague, as the operative specifics of curative language use are conceptualized in manifold ways. Against this background the present paper argued that language-oriented psychotherapy research can profit from a clarification of its theoretical foundations. For this purpose we developed a theoretical framework that specifies four components which allows for reconstructing the “architecture” and working principles of “talking cure models” and discussed five selected models in terms of the framework components.

In conclusion, the question arises to what extent the framework can be helpful for systematically describing “talking cure models” beyond the five models discussed. It is important to note that our decision to focus on five specific models went along with the decision to omit other approaches, e.g., comprehensive models like Lacanian psychoanalysis (Lacan, 2002) and classifications of verbal interventions (Gumz et al., 2017), or specific approaches like positive self-verbalizations (Meichenbaum, 1977), linguistic pain coping (Stephens et al., 2009), or imagination techniques (Sell et al., 2017). Moreover, the “talking cure models” selected for the paper call for critical discussion. The notion of catharsis, for example, was controversially debated in psychotherapy research. Many authors discarded the concept as modern myth (Binstock, 1973), often with reference to findings of aggression research implying that expression of aggressive emotions entails an increase of anger (Bushman, 2002), whereas others insisted on valuing the concept (e.g., Moreno, 1955; Carver et al., 1989; Greenberg, 2002) while proposing theoretical adjustments (Nichols and Efran, 1985; Kosmicki and Glickauf-Hughes, 1997). Similarly, the clinical significance of the inhibition model can be questioned, as its implication that verbalizing emotions reduces emotional intensity contradicts the finding that dealing with emotional content increases experiential intensity (e.g., Gendlin, 1997). Moreover, reviews on emotion work in psychotherapy tend to distinguish different types of emotional processes (e.g., Greenberg and Pascual-Leone, 2006) which suggests that the inhibition mechanism captures only one aspect of symbolic emotional processing (Messina et al., 2016). Regarding the other models, it needs to be emphasized that beyond the approaches focused here related approaches were formulated that suggest variations of thinking the “talking cure” in terms of symbolization, narrative, or metaphor. Gendlin (1997), for example, formulated a symbolization model which operates with a generalized notion of “felt meaning” that is not restricted to relational experiences. Furthermore, he defines symbols more broadly as anything that functions to “express, delineate, explicate, represent, conceptualize (other words are equally descriptive) the felt meaning” (Gendlin, 1997, p. 108). Similarly, therapeutic work with metaphors has been described in various terms, e.g., by Borbely (2009) who combines the concept of metaphor with the concept of metonymy to develop a complex notion of psychoanalytic processes. The same applies to narrative research that has given rise to different approaches (e.g., Boothe et al., 2005; Angus, 2012). By trend, most narrative models conceptualize narrative reorganization in more symmetric terms than Schafer, as “co-construction between client and therapist of an overarching and integrating narrative” (Grafanaki and McLeod, 1999, p. 297). Additionally, the notion of coherence as central objective of narrative “rebiographing” has been questioned by findings which suggest that qualities like complexity, diversity, dynamism, or polyphony are equally important for functional narratives (Avdi and Georgaca, 2007).

In summary, these considerations underscore our assertion that language-oriented psychotherapy research is characterized by a plethora of disparate “talking cure models” which advance different understandings of what “talking” means and what “cure” implies. If our framework as devised in this paper can cover all properties of this multitude of models needs to be investigated in further research. In the meantime, we are confident that the framework establishes a starting point for such endeavors by defining terms for components typically comprised in “talking cure models,” i.e., (1) a *foundational theory*, (2) an *experiential problem state*, (3) a *curative linguistic activity*, and (4) a *change-mechanism*. It can be used as terminological tool that allows for a clarification of the theoretical structure of “talking cure models” which, in turn, allows for the development of precise operationalizations, measurement techniques, and strategies to evaluate effects of linguistic activity (Russell, 1989) which can help to integrate findings of language-oriented psychotherapy research and establish a more comprehensive understanding of the mechanisms underlying curative language use within the complex multiplicity of factors involved in therapeutic change processes.

## Author contributions

CM had the idea for the paper, performed the acquisition, analysis, and interpretation of data for the work, and drafted the manuscript. CM and AG conceived the paper. CB participated in the design of the paper. AG coordinated the paper. AG and CB revised it critically for important intellectual content. All authors gave their final approval of the version to be published. All authors agreed to be accountable for all aspects of the work in ensuring that questions related to the accuracy or integrity of any part of the work are appropriately investigated and resolved.

### Conflict of interest statement

The authors declare that the research was conducted in the absence of any commercial or financial relationships that could be construed as a potential conflict of interest.

## Footnotes

^1^In the original German text Breuer and Freud use the term “eingeklemmter Affekt,” implying that the affect is “jammed” or “stuck” rather than “strangulated” (Breuer and Freud, 1895/2007, p. 40).
